# Practices, awareness, and perception towards home-based COVID-19 management among the general population in Mangalore city in south India

**DOI:** 10.12688/f1000research.74514.2

**Published:** 2022-03-18

**Authors:** Nitin Joseph, Vijay Pratap Singh, Impana Venkatesha Murthy, Vishaan Raman, Meera Banihatti Nagaraj, Rahul Vishwanath Shetty, Krishna Sai Vemuri, Shruthi Shreedhara, Maranakatte Shridhar Sumukha Manja

**Affiliations:** 1 Department of Community Medicine, Kasturba Medical College, Mangalore, Manipal Academy of Higher Education, Manipal, PIN code 575001, India; 2 Department of Physiotherapy, Kasturba Medical College, Mangalore, Manipal Academy of Higher Education, Manipal, PIN code 575001, India; 3 Kasturba Medical College, Mangalore, Manipal Academy of Higher Education, Manipal, PIN code 575001, India

**Keywords:** home-based management, practices, awareness, perception, general population, COVID-19

## Abstract

**Background: **Most patients with COVID-19 experience mild illness, which can be managed in a home environment. This study was done to assess the perception, awareness, and practices regarding home-based management of COVID-19 among the general population in India.

**Methods: **This cross-sectional study was done in May and June 2021. Data were collected using a Google Form.

**Results:** Mean age of the 294 participants was 36.6 ± 12.1 years. Of these participants, 45 (15.3%) were diagnosed with COVID-19 anytime in the past. Among them, 37 (82.2%) underwent home-based management for COVID-19. Monitoring body temperature and oxygen saturation were performed just once a day by 15.2% and 5.9% of COVID-19 patients respectively. Self-medication was practiced by 11 (29.7%) patients.

Disposable face masks were worn by 23 (62.2%) patients beyond eight hours of continuous usage. The disposable type of face mask was not discarded despite becoming wet, and cloth masks were worn by eight (21.6%) patients. Disposable gloves were only worn by 14 (37.8%) caregivers of COVID-19 patients. As many as 10 (27%) patients were tested for COVID-19 after completion of home isolation.

Awareness of all mild symptoms and signs of the disease was known to only 19 (6.5%) participants. Normal oxygen saturation in the blood was known to 40 (13.6%) participants. Just six (2%) participants were aware of the correct duration of home isolation in a symptomatic patient with COVID-19. The recommended duration of handwashing with soap and water was known to 102 (34.7%) participants.

As many as 17.4% and 32.7% of participants were not confident in using thermometer and pulse oximeter respectively.

**Conclusion:** Practices, awareness, and perception regarding certain essential measures in COVID-19 home management were found lacking among several participants. These need to be addressed by suitable training programs among the general population.

## Introduction

The coronavirus disease 2019 (COVID-19) is an emerging disease that continues to be a pandemic throughout the world. India is currently recovering from the second wave and is on the verge of a third wave in the months to come ( https://timesofindia.indiatimes.com/). During the second wave, the country faced a severe shortage of medical facilities and hospital beds ( https://reliefweb.int/). Fortunately, most patients with COVID-19 experience mild illness and can therefore be managed in the home setting (MoH & FW, 2021 ( https://www.mohfw.gov.in/); Public Health Unit, 2020 ( https://www.un.org/)). Apart from the commonly known clinical presentation of COVID-19, the various psychological problems among those affected by COVID-19 are gaining importance. A study carried out in China reported that anxiety disorders and depression are common among COVID-19 patients managed in the hospital settings. ^1^ A study in USA stated that anxiety disorders were not only common among COVID-19 patients but were also self-rated as severe. ^2^ Isolation of patients in a home environment with the support of family members may help to alleviate these psychological problems.

An Italian study reported that home management of COVID-19 patients following a standard prevention algorithm reduced the risk of hospitalization and thereby the cost of medical care by more than 90%. ^3^ On the contrary, if home management is not practised as per guidelines, the transmission of the disease to other household members could be disastrous. Monitoring of the vital parameters by patients or their caregivers and identifying and reporting of any danger signs helps in the timely hospital admission of patients developing complications during home isolation. Therefore, this study was done to assess the practices, perception, and awareness of home-based management of COVID-19 patients among the general population in an urban area in south India.

## Methods

This cross-sectional study was done during the second wave of COVID-19 in Mangalore city in May and June 2021. Mangalore is located between the Arabian sea and the Western Ghats, and it is the only city in Karnataka state, India, with all four modes of transport namely, road, rail, air, and sea. This makes the local population highly susceptible to COVID-19 infection.

The population of Mangalore city in the year 2021 is estimated to be 724,159 ( https://worldpopulationreview.com/). In May 2021, as many as 31,090 cases and 156 deaths due to COVID-19 were reported in Mangalore city, according to the records of Mangalore City Corporation.

In the study conducted in USA ^2^, 88.9% of COVID-19 patients undergoing home-based management monitored their blood pressure. Using this proportion, the sample size using the formula N = 4PQ/d ^2^ comes to 200 at 95% CI and 5% relative precision. Adding 20% as the non-response rate, the minimum sample size was taken as 240 participants. Data collection was carried out from May 29 ^th^, 2021 to June 7 ^th^, 2021. It was stopped after completing the stipulated data collection period of ten days.

Data were collected using an anonymous semi-structured questionnaire designed as a Google Form. ^4^ It was content validated with the help of a subject expert from the Department of Internal Medicine, Kasturba Medical College, Mangalore. It was later pilot tested among a group of ten people who were later excluded from the main study.

The link to the questionnaire was then circulated among the local population using WhatsApp. This was done to minimize exposure with the study participants during the data collection process in the background of the ongoing COVID-19 pandemic. The questionnaire is available as an extended data. ^4^

The study information sheet and the consent form constituted the first page of the questionnaire. Participants not willing to participate in this research study were given the option to decline consent leading to termination and submission of an empty questionnaire. Participants below 18 years of age were also excluded from this study.

The questionnaire was prepared using an extensive literature search. The websites of the World Health Organization, Centers for Disease Control and Prevention, Ministry of Health and Family Welfare Government of India, Indian Council of Medical Research, Government of Karnataka state, and previously published research articles related to the research topic were referred for preparing a comprehensive questionnaire. It had four sections to obtain information regarding socio demographic variables, practices, perception, and awareness of respondents regarding home-based COVID-19 management. Only one eligible participant per household was instructed to complete the questionnaire. This was possible because an instruction stating that only one member from the same household is supposed to fill this form was mentioned at the start of the questionnaire.

Socio economic status (SES) of participants were assessed using the Modified BG Prasad classification of 2020. ^5^

Submitted responses were extracted from the Google form entered in Microsoft Excel 2016, and transferred to SPSS version 25.0, Armonk, New York for data analysis.

The reliability of the data collection tool was calculated using Cronbach’s alpha value. Its value was calculated from the responses to questions and was found to be 0.81 indicating good internal consistency.

Approval was obtained from the institutional ethics committee of Kasturba Medical College, Mangalore before starting this study. The approval number was IEC KMC MLR 06-2021/205.

## Results

The mean age of the 294 participants was 36.6 ± 12.1 years. The majority of participants were female [160 (54.4%)] and the majority were graduates [132 (44.9%)] SES could be assessed among 165 participants. Among them, 87(52.7%) belonged to Class I SES ( Table 1).

**Table 1.** Sociodemographic distribution of study participants.

Characteristics	Number	Percentage
**Age group (years)**		
≤20	33	11.2
21–30	79	26.9
31–40	61	20.7
41–50	80	27.2
>50	41	14.0
**Gender**		
Males	134	45.6
Females	160	54.4
**Occupation**		
Unemployed/Retired	6	2.0
Student	66	22.5
Housewife	38	13.0
Semi-skilled	43	14.6
Skilled	48	16.3
Semi-professional	33	11.2
Professional	60	20.4
**Educational status**		
High school	28	9.5
Intermediate or Post high school diploma	94	32.0
Graduate	132	44.9
Postgraduate	40	13.6
**Type of family**		
Nuclear family	247	84.0
Joint family	33	11.2
Three generation family	14	4.8
**Socio-economic status (n = 165)**		
Class I	87	52.7
Class II	57	34.6
Class III	16	9.7
Class IV	5	3.0
**Nationality**		
Indian	279	94.9
Non-residential Indian	13	4.4
Foreigner	2	0.7
**Place of residence**		
Urban	216	73.5
Semi-urban	51	17.3
Rural	27	9.2
**Total**	**294**	**100.0**

Out of the total participants, 45 (15.3%) were diagnosed with COVID-19 at any time in the past. Among them, 32 (71.1%) were diagnosed using the RT-PCR test, five (11.1%) using the rapid antigen test, and eight (17.8%) using both the tests. One underwent a CT scan and was also tested with a D-dimer assay. The majority [30 (66.7%)] of COVID-19 diagnosed patients were females. Three patients were diagnosed with COVID-19 on more than one occasion. Usage of substances of abuse was reported among eight (17.8%) patients. Among them, seven were alcoholics, three were current smokers, and three were current tobacco chewers. Six (13.3%) patients had long-standing illnesses: all of them were hypertensive, three had diabetes mellitus, and two had cancer.

Out of the 45 patients with COVID-19, eight were managed at a hospital. The rest [37 (82.2%)] underwent home-based management for COVID-19. Reasons for hospital-based admission were due to the severity of the disease in five and due to a request by the patients to avoid the spread of the disease among other family members in three. Out of the five patients who had severe COVID-19, two developed complications. One had breathlessness, persistent cough, and very low oxygen saturation, while the other had telogen effluvium and developed adverse drug reactions.

The monitoring of body temperature and oxygen saturation was performed just once a day by 15.2% and 5.9% of COVID-19 patients respectively. Monitoring of respiratory rate and capillary refill time was practised by just 35.1% and 5.4% of patients respectively ( Table 2).

**Table 2.** Home-based screening and therapeutic care practices among participants who were diagnosed with COVID-19.

Home-based screening practices	Number	Percentage
**Monitoring of body temperature**	33	89.2
**Frequency of monitoring body temperature (n = 33)**		
Once a day	5	15.2
Twice a day	18	54.5
Three or more times a day	10	30.3
**Monitoring of oxygen saturation using a pulse oximeter**		
Yes	34	91.9
**Frequency of monitoring oxygen saturation in a day (n = 34)**		
Once	2	5.9
Twice	15	44.1
Three or more times a day	17	50.0
**Assessed lung functions test using breath-holding test**		
Yes	10	27.0
Monitoring of pulse rate	21	56.8
Monitoring of respiratory rate	13	35.1
Monitoring of capillary refill time	2	5.4
Maintained a logbook to record day to day readings	13	35.1
History of oxygen saturation dropping below the critical level (≤94%)	6	16.2
**Home-based therapeutic care practices**		
**Interventions performed to increase oxygen saturation (n = 6)** *		
Yoga exercises	6	100.0
Proning exercises	2	33.3
Yoga exercises (Pranayama) were effective to improve oxygen saturation (n = 6)	3	50.0
Proning exercises were effective to improve oxygen saturation (n = 2)	2	100.0
**Performed physical exercises during home isolation**	11	29.7
**Frequency of practising exercises (n = 11)**		
Daily	6	54.6
On alternate days	5	45.4
**Drugs used for management** *		
Paracetamol	33	89.2
Vitamin C	33	89.2
Cough syrup	15	40.5
Ivermectin	14	37.8
Vitamin D3	11	29.7
Cetirizine	9	24.3
Others ^†^	8	21.6
**The alternative system of medicine taken for COVID-19 management** *		
Ayurveda	6	16.2
Homeopathy	3	8.1
Unani	1	2.7
History of self-medication by COVID-19 patients	11	29.7
**Total**	**37**	**100.0**

* Multiple responses.

^†^ Others: Remdesivir 2, Favipiravir 2, Oral steroids 2, Doxycycline 1, Multivitamin syrup 1.

Systemic steroids were prescribed to two patients, and they were started on the first day of diagnosis. Self-medication was practised by 11 (29.7%) patients. The various drugs taken during self-medication were vitamin C tablets by five, paracetamol by four, multivitamin syrup by one and ivermectin tablets by one patient. Eight patients tried an alternative system for COVID-19 management. Among them, six tried Ayurveda. Three out of these six patients who tried Ayurvedic preparations for COVID-19 found it to be very effective ( Table 2).

Ten (27%) patients who underwent home isolation had to call their doctors for various reasons. The various reasons stated for telephonic consultation were to discuss the various symptoms experienced (by four), for associated fear (by one), for more information regarding self-monitoring practices (by one), to confirm the period of home isolation (by one), to enquire regarding medications to be taken (by two) and to enquire about medications to be continued after the completion of home isolation (by one).

All 37 patients undergoing home-based COVID-19 management used N95 or triple-layered surgical masks. However, 23 (62.2%) of them wore the disposable mask beyond eight hours of continuous usage. The disposable face mask was not discarded despite becoming wet by eight (21.6%) patients. At times, a cloth mask was worn by eight (21.6%) COVID-19 patients during home isolation. All 37 patients placed under home management had a single caregiver. Disposable gloves were worn by only 14 (37.8%) caregivers ( Table 3).

**Table 3.** Home-based preventive care practices among participants who were diagnosed with COVID-19.

Home–based preventive practices	Number	Percentage
Isolated in a separate room at home	37	100.0
The room used for isolation was well ventilated	37	100.0
A separate toilet facility was available	37	100.0
**Type of mask worn during the period of home isolation** *		
N95	24	64.9
Triple-layered surgical mask	19	51.3
Cloth mask	8	21.6
Single layered surgical mask	3	8.1
FFP2	2	5.4
Face shield	1	2.7
**Time after which the non-washable type of face mask was discarded after continuous usage**		
≤8 hours	14	37.9
8.1–24 hours	16	43.2
>24 hours	7	18.9
**The practice of immediately discarding the non-washable type of face mask in the event it became wet**		
Yes	29	78.4
**The practice of disinfecting the face mask before discarding**		
Yes	12	32.4
**Type of personal protective equipment worn by people taking care of COVID-19 patients during home-based management**		
N95	30	81.1
Triple-layered surgical mask	7	18.9
Disposable gloves	14	37.8
**Frequency of washing the cloth mask (n = 8)**		
More than once a day	1	12.5
Once a day	5	62.5
Once in two days	2	25.0
**Home remedies practised** *		
Took steam inhalation	32	86.5
Drank hot water fluids	31	83.8
Performed salt water gargles	28	75.7
Took more turmeric in their diet	22	59.5
Took more ginger in their diet	15	40.5
Took more garlic in their diet	10	27.0
Added Tulasi leaves in their diet	10	27.0
Performed salt and turmeric water gargles	9	24.3
Added honey to their diet	8	21.6
Medicated steam inhalation	7	18.9
Took protein-rich diet	1	2.7
**Practiced immersing laundry items in hot water before washing**		
Always	18	48.7
Sometimes	2	5.4
Never	17	45.9
**Frequently touched surfaces of COVID-19 patients undergoing home isolation were disinfected at least once a day**		
Yes	32	86.5
**Home visit by health care professionals**	12	32.4
**Type of professional attending for home visit (n = 12)** *		
Health workers	10	83.3
Government doctors/Medical officer	5	41.7
House surgeons	2	16.7
**Tested for COVID-19 after completion of home isolation**		
Yes	10	27.0
**Total**	**37**	**100.0**

* Multiple responses.

Thirty-two (86.5%) patients performed steam inhalation, and 11 (34.4%) of them reported that it was the most effective intervention providing relief compared to other conservative methods. Surfaces frequently touched by COVID-19 patients were not disinfected at least once a day by five (13.5%) patients or their caregivers ( Table 3).

Home visits by health care professionals were reported by 12 (32.4%) COVID-19 patients. The type of service rendered by personnel visiting the houses of COVID-19 patients was: monitoring of vital signs (by four), supply of medicines (by four), providing health education advice and home-based sanitization practices (by three), and performing clinical examination (by two) ( Table 3). As many as 10 (27%) patients were tested for COVID-19 after completion of home isolation ( Table 3).

Awareness of all the mild symptoms and signs of the disease was known to 19 (6.5%) participants. Two hundred and thirteen (72.4%) participants knew that home-based management is recommended for the mild stage of the disease. Normal oxygen saturation in the blood was known to 40 (13.6%) participants ( Table 4). Only six (2%) participants knew the correct duration of home isolation in a symptomatic patient with COVID-19. In terms of exercise, 235 (79.9%) had heard about proning, and the majority of them [166 (56.5%)] obtained this information from social media sources. Of all the participants, 209 (71.1%) had heard about COVID-19 self-testing kits. The recommended duration of handwashing with soap and water was known to 102 (34.7%) participants ( Table 5).

**Table 4.** Awareness of COVID-19 among the participants.

Characteristics	Number	Percentages
**Awareness of mild symptoms and signs of COVID-19** *		
Fever	182	61.9
Cough	137	46.6
Running nose	83	28.2
Sore throat	61	20.7
SpO _2_ more than 94%	19	6.5
**Awareness of moderate symptoms and signs of COVID-19** *		
Breathlessness	68	23.1
SpO _2_ between 90 to 94%	19	6.5
**Awareness of severe symptoms and signs of COVID-19** *		
Breathlessness	197	67.0
SpO _2_<90%	42	14.3
**Stage of COVID-19 in which home-based management is recommended**		
Mild stage	213	72.4
**Awareness of high-risk groups developing severe COVID-19** *		
Those with lung diseases	247	84.0
Those with poor immunity	246	83.7
Age above 60 years	211	71.8
Patients with diabetes mellitus	202	68.7
Those with cardiovascular diseases	175	59.5
Smokers	173	58.8
Those with cancer	115	39.1
Alcoholics	103	35.0
Those with kidney diseases	88	29.9
**Awareness of normal oxygen saturation in the blood**		
>94%	40	13.6
**Indicators of low oxygen level in the body** *		
Poor oxygen saturation displayed by the pulse oximeter	242	82.3
Cold hands and feet	190	64.6
Bluish discolouration of lips	172	58.5
Weak peripheral pulsation	170	57.8
Bluish discolouration of fingers	163	55.4
Low blood pressure	160	54.4
Bluish discolouration of the tongue	132	44.9
Delayed capillary refill time	94	32.0
Others ^†^	8	2.7
**Conditions wherein a patient undergoing home isolation should seek immediate medical attention** *		
Difficulty in breathing	256	87.1
Fall in oxygen saturation	245	83.3
Chest pain	187	63.6
Respiratory rate more than 30 cycles per minute	139	47.3
Mental confusion	107	36.4
Drowsiness	101	34.3
Bluish discolouration of body parts	57	19.4
**Total**	**294**	**100.0**

* Multiple responses.

^†^ Others: Numbness of hands and feet 2, Giddiness/Dizziness 2, Blackouts 1, Weakness 1, Hyperventilation 1, Confusion 1.

**Table 5.** Awareness of COVID-19 management practices among the participants.

Characteristics	Number	Percentages
**Period of home isolation in a COVID-19 patient with symptoms**		
Ten days from the date of the positive test result and thereafter three days as asymptomatic	6	2.0
**Period of home isolation in a COVID-19 patient without symptoms**		
Ten days from the date of positive test results	37	12.6
**Medication commonly used to relieve fever**		
Paracetamol	207	70.4
**Frequency of taking medication to relieve fever**		
Maintain a gap of four to six hours between doses	3	1.0
**Medications in COVID-19 management that should be started only after consulting a Doctor** *		
Remdesivir	234	79.6
Oral steroids	189	64.3
Inhalation steroids	180	61.2
**Heard of proning exercises**	235	79.9
**Source of information about proning exercises (n = 235)** *		
Social media	166	70.6
Doctor	97	41.3
Friends/Family/Relatives	88	37.4
WhatsApp	78	33.2
Television	78	33.2
Physiotherapist	38	16.2
Internet	2	0.8
Newspaper article	1	0.4
**Heard of breath-holding test to assess lung function**	236	80.3
**Heard about COVID-19 self-testing kits (Coviself)**	209	71.1
**Source of information about Coviself (n = 209)** *		
Social media	148	70.8
Television	86	41.1
Family/Friends/Relatives	70	33.5
WhatsApp	64	30.6
Newspaper	61	29.2
**Recommended duration of handwashing with soap and water**		
20 seconds	102	34.7
**Need of COVID-19 test after completion of home isolation**		
No	56	19.0
**Total**	**294**	**100.0**

* Multiple responses.

Out of the total participants, 210 (71.4%) had a thermometer, 195 (66.3%) had a pulse oximeter, 144 (49%) had a weighing machine, 128 (43.5%) had an electronic sphygmomanometer, 86 (29.2%) had a stethoscope, 40 (13.6%) had a mercury sphygmomanometer and 20 (6.8%) had an oxygen concentrator at their homes. As many as 17.4% and 32.7% of participants were not confident in using thermometer and pulse oximeter respectively ( Table 6).

**Table 6.** Perception of participants regarding various COVID-19 protective measures.

Characteristics	Number	Percentage
Confident in self-testing with Coviself self-testing kit (n = 209)	104	49.8
Confident in performing the procedure of recording body temperature using a thermometer	243	82.6
Confident in interpreting the results while using a thermometer	176	59.9
Confident in performing the procedure of recording oxygen saturation using a pulse oximeter	198	67.3
Confident in interpreting the results while using a pulse oximeter	158	53.7
Steam inhalation is an effective protective measure in COVID-19	196	66.7
Gargles with salt and turmeric are effective protective measures in COVID-19	178	60.5
**Breathing exercises can improve oxygen levels in the blood**		
Strongly agree	115	39.1
Agree	123	41.8
Neutral	39	13.3
Disagree	15	5.1
Strongly disagree	2	0.7
**Total**	**294**	**100.0**

## Discussion

### Screening and therapeutic care practices among COVID-19 patients

The proportion of participants who had previously had COVID-19 in this study was 15.3%. Among them, 66.7% were female, and 6.7% were current smokers. A Turkish study observed that 58.5% of COVID-19 patients were female, and 46.3% were current smokers. ^6^ It indicates that a greater proportion of female patients developed COVID-19.

In the present study, close to 90% of COVID-19 patients or their caregivers monitored blood oxygen saturation and body temperature. The frequency of monitoring body temperature was at least once a day among all patients. These findings were in accordance to the WHO framed international recommendations, which state that all COVID-19 patients isolated at home need to be monitored at least once a day for these vital signs ( WHO, 2021). Hence, the presence of a pulse oximeter and thermometer is essential for home-based COVID-19 care. However, monitoring of respiratory rate and capillary refill time was practised by just 35.1% and 5.4% of patients respectively in this study. Therefore healthcare workers need to take the initiative and train patients and their caregivers to measure these parameters daily ( MoH & FW, 2021). In a similar study done in USA, all COVID-19 patients managing their symptoms at home recorded their body temperature and oxygen saturation levels, 88.9% recorded their blood pressure and 55% their heart rate. ^2^ This means that if patients are trained, they can monitor their vital parameters and support self-care.

Approximately one-third of the patients in this study practised home-based exercises during home isolation, and more than half of them performed these exercises daily. This needs to be promoted in COVID-19 patients as physical activity at least once a day boosts the body’s immunity (Michigan Psychiatry Resource, 2021). In a Spanish study, 87% of COVID-19 patients found home-based exercises easy or very easy to perform. However, 73% of these patients contacted their physiotherapist, which was more frequent during the first week of isolation. ^7^ Physiotherapy in the home environment involving positioning the body, manoeuvres to help clear secretions in the respiratory tract, strengthening deconditioned muscle and de-stressing the mind is beneficial in COVID-19 care. It also teaches self-management techniques to help patients independently carry out their day-to-day activities. ^8^

In the present study, two patients who experienced a fall in oxygen saturation performed proning exercise and found it beneficial. Proning exercises improve breathing and oxygenation among patients facing a drop in oxygen saturation levels ( MoH & FW guidelines on proning, 2021).

More than one-third of the patients in this study during the home isolation were prescribed ivermectin and other antiviral drugs. In a Turkish study, 65.8% of COVID-19 patients in home-based care were taking anti-viral drugs, which was more than the observations of the present study. ^6^ This was against WHO guidelines which advise against antimicrobial treatment for patients with mild COVID-19 ( WHO, 2020). Two patients in this study were put on steroids within the first week of diagnosis of COVID-19. According to Indian government guidelines, systemic steroids are usually not indicated in mild COVID-19, and even if indicated, they need to be started beyond seven days of the onset of symptoms ( MoH & FW, 2021). An Italian study reported that steroids were prescribed in 30% of patients experiencing persistent symptoms of low oxygen saturation but after seven days following the onset of symptoms. ^3^ Therefore, guidelines for home-based treatment need to be strictly adhered to, and these aspects need to be emphasized during periodic training programmes for medical professionals at the setting.

Self-medication was practised by 29.7% of patients in this study. One of these patients had even self-medicated ivermectin. This was against the government recommendation, which states that the decision to start an anti viral drug is to be made only by a qualified medical professional ( MoH & FW, 2021). People therefore need to be educated about the hazards of self-medication as a part of home-based COVID-19 care.

None of the patients in this study experienced worsening of their health condition during home isolation compared to 9.8% in the Turkish study who were hospitalized following home-based COVID-19 management. ^6^

### Preventive care practices among COVID-19 patients

All patients under home isolation reported that they were placed in a well-ventilated room. This ensures adequate cross ventilation as per the government recommendations ( MoH & FW, 2021). In the present study, the majority of participants wore N95 face masks followed by triple-layered surgical masks. However, about one-fourth of the patients (21.6%) at times also reported that they wore a cloth-type mask during home isolation. This is a dangerous practice as cloth masks are not as effective as disposable masks to prevent virus transmission. According to government guidelines, COVID-19 positive patients should always wear at least a triple layer surgical mask during the home isolation period ( MoH & FW, 2021). As many as 62.1% of the patients undergoing home isolation in this study wore a disposable mask beyond eight hours of continuous usage. According to recommendations, disposable masks must be discarded after eight hours of continuous use and earlier if soiled or wet ( MoH & FW, 2021; Bhatia, 2021). These masks should be disinfected with chemicals like 1% sodium hypochlorite before disposal ( MoH & FW, 2021). This is another area where information needs to be provided.

All 37 patients undergoing home care in this study had a single caregiver. This is in accordance with government recommendations where only one person in the family should be allocated as a caregiver ( Bhatia, 2021). Among the caregivers, 81.1% wore N95 masks, and the rest 18.9% wore a triple-layered surgical mask. However only 37.8% of them wore disposable gloves. As per the government recommendations, caregivers should wear a double mask or an N95 and should maintain a one-metre distance or more while taking care of COVID-19 patients ( Bhatia, 2021).

More than three-quarters of the patients in this study took steam inhalation, drank hot fluids, and performed saltwater gargles. As per the national recommendations, steam inhalation and performing warm water gargles are desirable twice a day in COVID-19 care ( MoH & FW, 2021).

The practice of immersing laundry items in hot water before washing was followed on every occasion by 48.7% of patients. As per WHO recommendations, clothes, bed linen, and bath towels of COVID-19 patients need to be immersed in water at 60 to 90 °C, washed with soap or detergent, and later dried thoroughly ( WHO & UNICEF, 2020). As many as 86.5% of patients or their caregivers in this study practiced disinfection of frequently touched surfaces in their houses at least once a day in accordance with WHO guidelines ( WHO & UNICEF, 2020). Disinfectants like 0.1% sodium hypochlorite need to be used for disinfecting surfaces.

Home visits by health care professionals were reported by close to one-third of COVID-19 patients in this study. They act as the primary point of contact between patients and health care facilities. This helps address immediate concerns among patients that have not been resolved during telephone conversations. Provision of a contact number of a health caregiver or establishment, to every patient under home isolation is a prerequisite in home-based COVID-19 management, as per national guidelines ( MoH & FW, 2021). Community health workers can monitor and train patients or their caregivers to monitor vital signs and recognize danger signs of poor oxygen saturation during home visits ( https://www.cdc.gov/coronavirus/). They also have an opportunity to offer psychological and social support to both patients and their families with the help of various training modules ( ICMR, 2021). This will help reduce the burden on the local health care delivery system. According to national guidelines, the district health authorities need to ensure that all patients under home isolation in their jurisdiction are strictly monitored ( MoH & FW, 2021). Home-based management for mild COVID-19 was reported to be safe and effective in a study in Pakistan. The study reported that by close monitoring, particularly of oxygen saturation with a pulse oximeter, the hospitalization rate among the home isolated patients was minimal, and there was no incident of any mortality. ^9^

More than one-quarter of the patients were tested for COVID-19 after completion of home isolation in this study. In comparison, in a Turkish study, a COVID-19 PCR test was repeated among 87.8% of patients undergoing home isolation. ^6^ However, according to Indian government recommendations, there is no need to re-test patients after completion of the suggested period of home isolation. This should be made known to all health caregivers ( MoH & FW, 2021).

### Awareness of COVID-19 and its management among participants

Symptoms of upper respiratory tract infection without breathlessness or without a drop in normal oxygen saturation in COVID-19 positive patients suggest a mild form of this disease ( MoH & FW, 2021). This fact was known only to 6.5% of the total participants in this study.

The risk factors of the severe form of COVID-19 as stated in literature are age above 60 years, an immunosuppressive state, the presence of co-morbidities like diabetes mellitus, hypertension, cancer, cerebrovascular and cardiovascular disease, chronic lung and renal diseases, and habits like smoking and alcohol consumption ( WHO Scientific Brief, 2020). In a study done in Iraq, among severe COVID-19 patients managed in a home environment, as many as 72.5% had at least one co-morbidity. ^10^

Less than 40% of the participants in this study were aware that the presence of cancer and renal diseases and being alcoholic are risk factors for the severe form of COVID-19. None of the participants were aware that the presence of cerebrovascular disease was also a risk factor for developing severe COVID-19.

The national and international guidelines state that medical attention needs to be given immediately if a patient with COVID-19 on home isolation experiences: a drop in normal oxygen saturation, difficulty in breathing, chest pain, mental confusion, feels drowsy, develops bluish discolouration of nail beds, lips or skin ( MoH & FW, 2021; CDC, 2021). The majority of the participants in this study were unaware that mental confusion, drowsiness, and bluish discolouration of body parts are red flags for hospital admission in COVID-19 patients placed under home isolation. Patients and their caregivers need to be made aware of these symptoms.

Every COVID-19 patient needs to isolate for 10 days after symptom onset, followed by three days without symptoms ( MoH & FW, 2021). This fact was known to a mere 2% of the participants in this study. Awareness of this guideline is essential to prevent the transmission of COVID-19 in the community.

More than three-quarters of participants in the present study had heard about proning exercises. More than seventy percent of them obtained this information from social media sources. Videos have been extensively circulated and used to train people in COVID-19 care. ^11^ This method can be used to train patients to perform proning, other chest expansion, and breathing exercises and active limb exercises. ^7^ This training approach has been successful in helping people perform these exercises correctly. ^7^

According to international guidelines, the recommended duration of hand washing with soap and water for 20 seconds was known to little more than one-third of the participants in this study ( Mayoclinic, 2021). This is an essential information for people to learn. This should therefore be emphasized during any awareness sessions held in the community.

The need for COVID-19 re-testing after completing home isolation is not recommended as per state guidelines ( Government of Karnataka, 2020). This fact was known to only 56 (19%) participants in this study. Along with the health caregivers, the patients also need to be made aware of this to minimize anxiety and unnecessary wastage of resources like COVID-19 testing kits.

### Perception of participants regarding COVID-19 protective measures

The confidence in performing and interpreting results of body temperature and blood oxygen saturation recording were found lacking among several participants in this study. This also needs to be addressed in future training programmes.

## Strengths of this study

This study has comprehensively assessed the practices, awareness, and perception among the general population regarding home-based management in COVID-19. It has highlighted several grey areas that need to be addressed by local health authorities and policymakers. 

## Limitations of this study

The participants in this study were enrolled using an online selection method considering the prevailing pandemic situation. Therefore, the sample chosen may not represent the entire population of Mangalore. There is also a possibility of non-reporting of information by the participants in this study.

## Conclusion

Practices, awareness, and perception regarding certain essential measures in COVID-19 home management were found lacking among several participants in this study. The local health authorities need to be address these inadequacies by implementing suitable training programmes among the general population in this setting.

## Data availability

### Underlying data

Figshare: ‘Practices, awareness and perception towards home-based COVID-19 management among the general population in south India’. https://doi.org/10.6084/m9.figshare.17057183. ^4^

This project contains the following underlying data:

• Home-based COVID care.sav
• Research data.xlsx

### Extended data

Figshare: ‘Practices, awareness and perception towards home-based COVID-19 management among the general population in south India’. https://doi.org/10.6084/m9.figshare.17057183. ^4^

This project contains the following extended data:

• Questionnaire

Data are available under the terms of the Creative Commons Attribution 4.0 International license (CC-BY 4.0)

## Consent

Written informed consent for publication of the participants’ details was obtained from the participants.
