# Supplementary material for: Practices, awareness, and perception towards home-based COVID-19 management among the general population in Mangalore city in south India
Source: F1000Res. 2022 Mar 18;10:1271. Originally published 2021 Dec 10. [Version 2] doi: 10.12688/f1000research.74514.2 (PMC8943345; doi:10.12688/f1000research.74514.2)
Supplement: Peer review history

# Peer review: Reviewer response for version 2

**DOI:** 10.5256/f1000research.122366.r127915

**Published:** 2022-03-23

**Authors:** Pathiyil Ravi Shankar

**Affiliations:** 1 IMU Centre for Education, International Medical University, Kuala Lumpur, Malaysia

The authors have addressed my comments.

Is the work clearly and accurately presented and does it cite the current literature?

Yes

If applicable, is the statistical analysis and its interpretation appropriate?

Yes

Are all the source data underlying the results available to ensure full reproducibility?

Yes

Is the study design appropriate and is the work technically sound?

Yes

Are the conclusions drawn adequately supported by the results?

Yes

Are sufficient details of methods and analysis provided to allow replication by others?

Partly

Reviewer Expertise:

Rational use of medicines, COVID-19, medical education

I confirm that I have read this submission and believe that I have an appropriate level of expertise to confirm that it is of an acceptable scientific standard.

# Peer review: Reviewer response for version 2

**DOI:** 10.5256/f1000research.122366.r127916

**Published:** 2022-03-21

**Authors:** Ganesh Kumar Saya

**Affiliations:** 1 Department of Preventive and Social Medicine, Jawaharlal Institute of Postgraduate Medical Education and Research (JIPMER), Puducherry, India

All comments have been addressed.

Is the work clearly and accurately presented and does it cite the current literature?

Yes

If applicable, is the statistical analysis and its interpretation appropriate?

Yes

Are all the source data underlying the results available to ensure full reproducibility?

Yes

Is the study design appropriate and is the work technically sound?

Yes

Are the conclusions drawn adequately supported by the results?

Yes

Are sufficient details of methods and analysis provided to allow replication by others?

Yes

Reviewer Expertise:

Mental health, Maternal and Child health

I confirm that I have read this submission and believe that I have an appropriate level of expertise to confirm that it is of an acceptable scientific standard.

# Peer review: Reviewer response for version 1

**DOI:** 10.5256/f1000research.78279.r122509

**Published:** 2022-02-11

**Authors:** Pathiyil Ravi Shankar

**Affiliations:** 1 IMU Centre for Education, International Medical University, Kuala Lumpur, Malaysia

This is an important study carried out among individuals in Mangalore, South India on practices, awareness, and perceptions regarding home-based COVID management. The study presents new information but does have the limitation of online selection of a limited number of respondents. The respondents selected may or may not be reflective of the general population. My specific comments are shown below.   

- Methods section: I did not understand the mention of the use of monitoring blood pressure in a United States study to calculate the sample size.
- The repetition between the Results and the Discussion can be reduced. The authors can share the questionnaire used in the appendix.
- The method of questionnaire development should be mentioned in greater detail.
- Are patients briefed about the criteria mentioned and the guidelines to be followed before they undergo home quarantine?
- The title should be modified to reflect the fact that the study was conducted only in one city in South India and may not reflect the entire population of the region.   
- The standard of written English is good but may require some modifications in certain areas. Are there more studies dealing with home-based COVID management in the literature?
- The authors can consider the use of long paragraphs. There are many lone sentences in the manuscript.
- At the end of the Discussion, the authors should add a section on the strengths and limitations of the study.

Is the work clearly and accurately presented and does it cite the current literature?

Yes

If applicable, is the statistical analysis and its interpretation appropriate?

Yes

Are all the source data underlying the results available to ensure full reproducibility?

Yes

Is the study design appropriate and is the work technically sound?

Yes

Are the conclusions drawn adequately supported by the results?

Yes

Are sufficient details of methods and analysis provided to allow replication by others?

Partly

Reviewer Expertise:

Rational use of medicines, COVID-19

I confirm that I have read this submission and believe that I have an appropriate level of expertise to confirm that it is of an acceptable scientific standard, however I have significant reservations, as outlined above.

# Response

**Published:** 2022-03-11

**Authors:** Nitin Joseph

**Affiliations:** Kasturba Medical College, Mangalore, Manipal Academy of Higher Education, Manipal, India, India

**Original comments of the reviewer:** The study has the limitation of online selection of a limited number of respondents. The respondents selected may or may not be reflective of the general population.

**Reply by the author(s)**: This statement has been added under the limitation section.

**Original comments of the reviewer:** I did not understand the mention of the use of monitoring blood pressure in a United States study to calculate the sample size.

**Reply by the author(s): **One of the study objectives was to assess the practices of patients during the home management of COVID-19. One of the essential practices recommended among COVID-19 patients is the monitoring of vital parameters on a day-to-day basis during home management. Hence, the proportion of patients monitoring their blood pressure in a study done in USA was used to calculate the sample size in the present study.

**Original comments of the reviewer**: The repetition between the Results and the Discussion can be reduced.

**Reply by the author(s):** The last paragraph under the discussion section, which was a repetition of the results section, has been completely deleted.

**Original comments of the reviewer:** The authors can share the questionnaire used in the appendix.

**Reply by the author(s):** Unfortunately, F1000 do not offer appendices on their articles, so the questionnaire is supplied as extended data.

**Original comments of the reviewer: **The method of questionnaire development should be mentioned in greater detail.

**Reply by the author(s):** In order to make a comprehensive questionnaire, several websites of national and international bodies along with previously published research articles related to the research topic were referred by the investigators. The details of the same have been added under the methods section as suggested.

**Original comments of the reviewer:** Are patients briefed about the criteria mentioned and the guidelines to be followed before they undergo home quarantine?

**Reply by the author(s): **Yes, all patients under home isolation or home quarantine are briefed at the beginning about the criteria mentioned and the guidelines to be followed during the home isolation period.

**Original comments of the reviewer: **The title should be modified to reflect the fact that the study was conducted only in one city in South India and may not reflect the entire population of the region.

**Reply by the author(s):** The title has been changed as per the suggestion.

**Original comments of the reviewer: **The standard of written English is good but may require some modifications in certain areas. The authors can consider the use of long paragraphs. There are many lone sentences in the manuscript.

**Reply by the author(s): **The entire manuscript has been language edited as per the suggestion. The paragraph breaks have also been edited as per the suggestion.

**Original comments of the reviewer: **Are there more studies dealing with home-based COVID management in the literature?

**Reply by the author(s): **Findings from two recent studies on home-based COVID-19 care done in two developing countries in Asia have been added to the discussion section. (Reference 9 and 10)

**Original comments of the reviewer:** At the end of the Discussion, the authors should add a section on the strengths and limitations of the study.

**Reply by the author(s): **These sections have been added at the end of the discussion section as suggested.

# Peer review: Reviewer response for version 1

**DOI:** 10.5256/f1000research.78279.r102501

**Published:** 2022-01-10

**Authors:** Ganesh Kumar Saya

**Affiliations:** 1 Department of Preventive and Social Medicine, Jawaharlal Institute of Postgraduate Medical Education and Research (JIPMER), Puducherry, India

In light of the ongoing 3 ^rd^ wave of COVID-19 which is being witnessed in most parts of the world, this study is very relevant and well-written in all the parts of the study. The majority of patients currently infected with Omicron have mild illness and are recommended isolation in a home environment. This research study brings out the current understanding of the general population regarding steps to be followed during home isolation for COVID-19 management and the grey areas that need to be addressed by policymakers and health care providers.

**Some comments:**

- Introduction -1st sentence may be modified as - possible verge of the start of a third wave.
- How one member from a family is selected may be mentioned in the methods section.

Is the work clearly and accurately presented and does it cite the current literature?

Yes

If applicable, is the statistical analysis and its interpretation appropriate?

Yes

Are all the source data underlying the results available to ensure full reproducibility?

Yes

Is the study design appropriate and is the work technically sound?

Yes

Are the conclusions drawn adequately supported by the results?

Yes

Are sufficient details of methods and analysis provided to allow replication by others?

Yes

Reviewer Expertise:

Mental health, Maternal and Child health

I confirm that I have read this submission and believe that I have an appropriate level of expertise to confirm that it is of an acceptable scientific standard.

# Response

**Published:** 2022-03-11

**Authors:** Nitin Joseph

**Affiliations:** Kasturba Medical College, Mangalore, Manipal Academy of Higher Education, Manipal, India, India

**Original comments of the reviewer: **1st sentence may be modified as - possible verge of the start of a third wave

**Reply by the author(s): **The third wave of COVID-19 started in the last week of November 2021 and continued to rise thereafter. Currently, in March 2022, there is a decline in the daily number of COVID-19 new cases. Hence, the statement has been left as it is. 

**Original comments of the reviewer:** How one member from a family was selected may be mentioned in the methods section.

**Reply by the author(s):** An instruction stating that only one member from the same household is supposed to fill this form was mentioned at the start of the questionnaire.
